# Characterization and evaluation of antimicrobial and cytotoxic effects of *Streptomyces* sp. HUST012 isolated from medicinal plant *Dracaena cochinchinensis* Lour.

**DOI:** 10.3389/fmicb.2015.00574

**Published:** 2015-06-08

**Authors:** Thi-Nhan Khieu, Min-Jiao Liu, Salam Nimaichand, Ngoc-Tung Quach, Son Chu-Ky, Quyet-Tien Phi, Thu-Trang Vu, Tien-Dat Nguyen, Zhi Xiong, Deene M. Prabhu, Wen-Jun Li

**Affiliations:** ^1^Key Laboratory of Microbial Diversity in Southwest China, Ministry of Education, Yunnan Institute of Microbiology, Yunnan UniversityKunming, China; ^2^Department of Food Technology, School of Biotechnology and Food Technology, Hanoi University of Science and TechnologyHanoi, Vietnam; ^3^Key Laboratory for Forest Resources Conservation and Use in the Southwest Mountains of China, Ministry of Education, Southwest Forestry UniversityKunming, China; ^4^State Key Laboratory of Biocontrol, Key Laboratory of Biodiversity Dynamics and Conservation of Guangdong Higher Education Institutes, College of Ecology and Evolution, Sun Yat-Sen UniversityGuangzhou, China; ^5^Laboratory of Fermentation Technology, Institute of Biotechnology, Vietnam Academy of Science and TechnologyHanoi, Vietnam; ^6^Department of Bioactive Products, Institute of Marine Biochemistry, Vietnam Academy of Science and TechnologyHanoi, Vietnam

**Keywords:** endophytic, *Streptomyces* sp. HUST012, *Dracaena cochinchinensis* Lour., antimicrobial and cytotoxic activities, (*Z*)-tridec-7-ene-1,2,13-tricarboxylic acid, Actinomycin-D

## Abstract

A highly potent secondary metabolite producing endophytic strain, *Streptomyces* sp. HUST012 was isolated from the stems of the medicinal plant *Dracaena cochinchinensis* Lour. Strain HUST012 showed antimicrobial and antitumor activities which were significantly much higher than those of dragon's blood extracted from *D. cochinchinensis* Lour. On further analysis, the strain was found to produce two metabolites, SPE-B11.8 (elucidated to be a novel metabolite (*Z*)-tridec-7-ene-1,2,13-tricarboxylic acid) and SPE-B5.4 (elucidated as Actinomycin-D). The Minimum Inhibitory Concentration values of SPE-B11.8 against a set of test bacterial organisms (Methicillin-resistant *Staphylococcus epidermis* ATCC 35984, Methicillin-resistant *Staphylococcus aureus* ATCC 25923, *Escherichia coli* ATCC 25922, and *Klebsiella pneumoniae* ATCC 13883) ranged between 15.63 and 62.5 μg/ml while that for SPE-B5.4 ranged between 0.04 and 2.24 μg/ml. The compound SPE-B11.8 showed cytotoxic effect at 41.63 and 29.54 μg/ml I*C*_50_-values against Hep G2 and MCF-7, respectively, while the compound SPE-B5.4 exhibited stronger activities against them at 0.23 and 0.18 μg/ml I*C*_50_-values.

## Introduction

*Streptomyces* spp. have been shown to possess the ability to synthesize antibacterial, antifungal, insecticidal, antitumor, anti-inflammatory, anti-parasitic, antiviral, anti-infective, antioxidant, and herbicidal compounds (Qin et al., 2011; Kawahara et al., 2012). Nearly 70% of the natural antibiotics used in clinical practices were produced by actinobacteria (Subramani and Aalbersberg, 2012) of which 75–80% have been derived from *Streptomyces* alone (Inbar and Lapidot, 1988; Olano et al., 2004; Rehm et al., 2009; Crnovcic et al., 2013).

The plant *Dracaena cochinchinensis* Lour. has been used as a traditional medicine since ancient times in the form of Dragon's blood, a deep red resin. Dragon's blood has been shown to illustrate antimicrobial, antiviral, antitumor, cytotoxic, analgesic, antioxidative, anti-inflammatory, haemostatic, antidiuretic, anti-ulcer and wound healing activities (Gupta et al., 2008). It also finds application as coloring materials and wood varnish (Gupta et al., 2008). However, the slow growth in combination with its low dragon's blood yield results in the destruction of large number of century old plant for harvesting a few milligrams of dragon's blood (Fan et al., 2008). This current study was conducted to explore a sustainable way of utilizing the medicinal plant by studying the endophytic actinomycetes associated with the plant. This paper incorporated the results of the characterization and the evaluation of cytotoxic and antimicrobial effects of an endophytic *Streptomyces* sp. strain, isolated from the medicinal plant *D. cochinchinensis* Lour. in comparison with those of dragon's blood extracted from the host plant. The paper also reported the structure elucidation of the bioactive metabolites extracted from the endophytic actinobacteria.

## Materials and methods

### Sample collection and isolation of endophytic actinomycete

Healthy stems of *D. cochinchinensis* Lour. plant were collected from Cuc Phuong National Park, Ninh Binh province, Vietnam (20° 19′ 8″N, 105° 37′ 20″E; 338 m). The samples were surface sterilized and plated on Sodium propionate medium (Qin et al., 2009). The medium was supplemented with nalidixic acid (25 mg/l), nystatin (50 mg/l), and K_2_Cr_2_O_7_ (50 mg/l) to inhibit the growth of Gram-negative bacteria and fungi and polyvinyl pyrolidone (PVP) 2% and tannase 0.005% to improve the growth of colonies. Actinomycetes colonies grown on this culture media were selected and purified by repeated streaking onto International *Streptomyces* Project (ISP) 2 medium. The purified strain HUST012 was preserved as glycerol suspensions (20%, v/v) and as lyophilized spore suspensions in skim milk at −80°C (Zhang et al., 2010).

### Characterization of the endophytic isolate HUST012

The endophytic isolate HUST012 was characterized on the basis of the physiological and biochemical properties and the analysis of 16S rRNA gene sequence. Morphological and growth patterns were observed on different media (Shirling and Gottlieb, 1966). Morphological characteristics were observed by light microscopy (Olympus BH2) and scanning electron microscopy (JSM-6610LV, JEOL Ltd.) (Anderson and Wellington, 2001). The ability of the isolate to grow at different pH (4.0–10.0, at intervals of 1.0 pH unit using the buffer system as described by Xu et al., 2005) and concentration of NaCl (0, 0.5, 1.0, 2.0, 3.0, 4.0, 5.0, 6.0, 7.0, 10.0, 11.0, 12.0, 15.0%, w/v) was examined on ISP 2 medium. Growth was tested at 4, 10, 20, 25, 28, 37, 45, and 55°C using ISP2 medium. The hydrolysis of starch, casein and gelatin was carried out according to the methods described by Tindall et al. (2007). Nitrate reduction and H_2_S production were determined using conventional procedures (Goodfellow, 1971; Athalye et al., 1985). Utilization of the carbon source was performed as previously described (Shirling and Gottlieb, 1966; Athalye et al., 1985; Mechri et al., 2014) using the basal medium recommended by Pridham and Gottlieb (1948).

The isolation of genomic DNA and PCR amplification for 16S rRNA gene was performed as previously described (Li et al., 2009). The identification of phylogenetic neighbors and calculation of pairwise 16S rRNA gene sequence similarities were achieved using the EzTaxon server (http://www.eztaxon.org/) (Kim et al., 2012) and BLAST analysis (http://blast.ncbi.nlm.nihgov/Blast.cgi). Multiple sequence alignment was done using CLUSTALW (Thompson et al., 1997). The phylogenetic tree was constructed using the aligned sequences by the neighbor-joining method (Saitou and Nei, 1987) using Kimura-2-parameter distances (Kimura, 1983) in the MEGA 6 software (Tamura et al., 2013). To determine the support of each clade, bootstrap analysis was performed with 1000 replications (Felsenstein, 1985).

The GenBank accession number for the partial 16S rRNA gene sequences of strain HUST012 is KP330557.

### Evaluation of antimicrobial activities

The antibacterial activities was evaluated against Methicillin-susceptible *Staphylococcus aureus* (MSSA) ATCC 29213, Methicillin-resistant *Staphylococcus epidermidis* (MRSE) ATCC 35984, Methicillin-resistant *Staphylococcus aureus* (MRSA) ATCC 25923, *Klebsiella pneumoniae* ATCC 13883, *Aeromonas hydrophila* ATCC 7966, *Escherichia coli* ATCC 25922, *Escherichia coli* ATCC 11105, and *Enterococcus faecalis* ATCC 29212 using the agar well diffusion method (Holder and Boyce, 1994). The Minimum Inhibitory Concentration (MIC) was determined as previously described (Andrews, 2001).

The animal fungal pathogens *Fusarium graminearum*, *Aspergillus carbonarius*, and *Aspergillus westerdijkiae* which were known to produce strong toxic deoxynivalenol (DON) and ochratoxin A (Khamna et al., 2009; Huffman et al., 2010) were kindly provided by UMR Qualisud, CIRAD, France. These strains were maintained on Potato Dextrose Agar (PDA) medium (Liu et al., 2002). For the determination of antifungal activity, culture broth of HUST012 (100 ml) was centrifuged at 7000 *g* for 10 min. The supernatant was collected and added to the PDA medium (pH 5.5) at a concentration of 15% (v/v). Sterilized water was used as control. The resulting PDA plates were inoculated with the different fungal strains and incubated for 5 days at 28°C. The fungal radial growth was measured. Each experiment was carried out in triplicates.

### Determination of cytotoxic activity

The cytotoxicity against human hepatocellular carcinoma Hep G2 and human breast adenocarcinoma MCF-7 cell lines was tested by using sulforhodamine B (SRB) assay as previously described (Thao et al., 2014). Ellipcitine was used as the positive control. The test was done in triplicates to ensure accuracy.

### Fermentation

A small-scale liquid fermentation was performed with YIM 61 medium (Qin et al., 2009) as the antibiotic producing medium (200 rpm, 28°C, 5 days). The scale up fermentation (20 L) was done using the New Brunswick BioFlo®/CelliGen® 115 Benchtop Fermentor & Bioreactor (28°C, 5 days). In both cases, seed culture for inoculation was prepared in ISP2 medium (200 rpm, 28°C, 4 days)

### Extraction and purification of the active compounds

The fermentation broth was centrifuged at 7000 rpm for 10 min. The supernatant fraction was then extracted thrice with ethyl acetate. The ethyl acetate layer was concentrated *in vacuo* to give ethyl acetate extract (SPA-E). The aqueous phase was filtered through a diaion HP20 column and eluted with water and methanol subsequently. The water eluent fraction was evaporated to give the extract designated as SPA-W1 while the methanol eluent was concentrated *in vacuo* to obtain a brown solid (SPA-W2).

**Figure 1:**
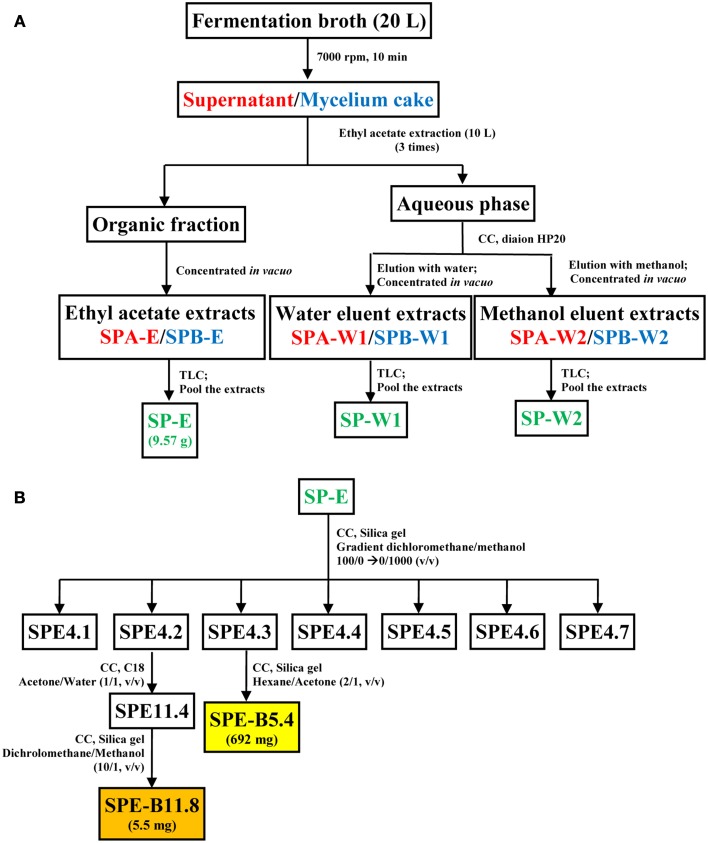
**Schematic representation of the process for metabolite extraction from strain HUST012. (A)** Extraction of the fermentation medium into crude metabolite extracts; **(B)** fractionation protocol for pure compounds SPE-B11.8 and SPE-B5.4 from the crude ethyl acetate extract. Note: CC, Column chromatography; Font in red color indicates supernatant, blue mycelium and green pooled fractions; Color boxes indicate pure compound.

Similarly, the mycelium cake obtained after centrifugation of the fermentation broth was processed to obtain a ethyl-acetate extract (SPB-E), a water eluent extract (EPB-W1) and a methanol eluent extract (SPB-W1). All these fractions were analyzed in Silica gel TLC sheet (Merck, Germany) using the dichloromethane-methanol (30:1, v/v) solvent system. Based on the similarity profiles in the TLC (Koup et al., 1978), SPA-E/SPB-E, SPA-W1/SPB-W1, and SPA-W2/SPB-W2 were pooled together and were designated as SP-E, SP-W1, and SP-W2, respectively. A schematic diagram representing the extraction protocol is shown in Figure 1. Each of these fractions was evaluated for antimicrobial and cytotoxic activities. The bioactive fractions were further purified using different solvent systems to obtain pure metabolite(s) as represented in Figure 1B.

### Structure elucidation of the pure active compounds

The structure of the bioactive compound(s) was analyzed using mass spectrometry (MS) and nuclear magnetic resonance (NMR) spectroscopy (Booth et al., 1976; Hamza et al., 2013). The results were compared with the available reference compounds and published literatures.

### Determination of antibacterial and cytotoxic effects of the dragon's blood extracted from medicinal plant *D. cochinchinensis*

Dragon's blood in the xylem of the host plant was extracted as described by Wang et al. (2011). The dry weight of the extract was dissolved in 95% (v/v) alcohol and filtered through sterile filter membrane (0.22 μm). The solution was then used for antibacterial and antitumor tests.

The MIC of Dragon's blood against MSSA ATCC 29213, MRSE ATCC 35984, *K. pneumoniae* ATCC 13883, and *E. coli* ATCC 25922 was determined by broth dilution method on 96-well plate as previously described (Andrews, 2001). The MIC against *F*. *graminearum* was determined according to Gopal et al. (2012). The SRB assay was used for determination the cytotoxic effect of the Dragon's blood on human breast adenocarcinoma (MCF-7) and human hepatocellular carcinoma (Hep G2) cells (Thao et al., 2014).

## Results

### Characterization of strain HUST012

Cells of the strain HUST012 was Gram-positive and aerobic. The strain formed extensively branched, non-fragmented substrate and aerial mycelia. Strain HUST012 formed straight or rectiflexibile spore chains with smooth surface. However, these spore chains generally contained less than 50 spores (Figure S1). The strain grew well on ISP 2, ISP 3, ISP 5, TSA, Czapek, and Nutrient agar media, with a gray color aerial mycelium. It produced green-yellow and yellow pigments on ISP2 and Czapek agar media respectively. Strain HUST012 was found to grow over a wide range of temperature (4–45°C) and pH (4.0–9.0) with optimal growth at 28°C and pH 6.0–7.0, and in the presence of upto 10% NaCl (w/v) with optimum at 1–3% NaCl.

**Figure 2:**
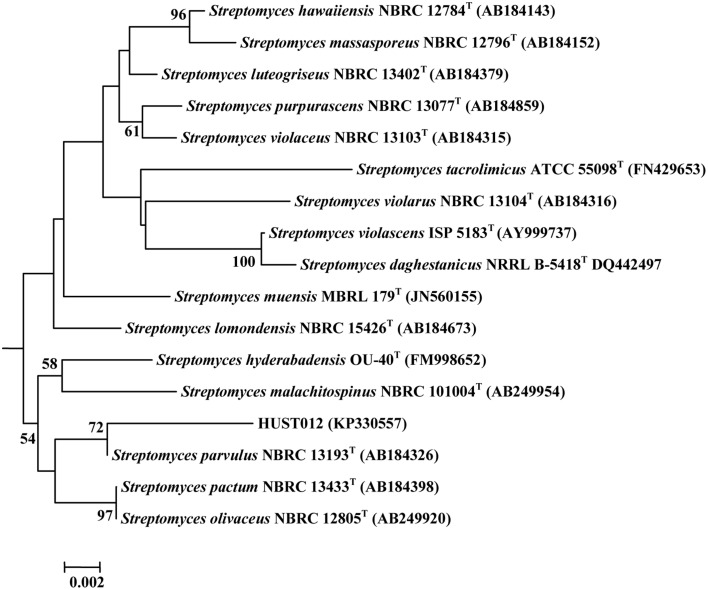
**Neighbor-joining tree showing the phylogenetic relationships based on 16S rRNA gene sequence of the strain HUST012 and closest species**. Bootstrap values (expressed as percentages of 1000 replications) greater than 50% was given at the node.

HUST012 could utilize DL-alanine, L-arginine, L-asparagine, Glycine, DL-leucine, L-lysine, DL-serine, L-glutamic acid, DL-methionine, L-cystine, L-histidine as nitrogen resources; D-fructose, D-galactose, D-glucose, D-mannose, D-trehalose, D-sorbose, D-xylose, glycerol, and sodium acetate as carbon sources. The strain was positive for amylase and catalase activities, but was negative for nitrate reduction, H_2_S production and gelatin reduction tests. Strain HUST012 showed highest 16S rRNA gene sequence similarities with *Streptomyces parvulus* (99.26%). Phylogenetic tree (Figure 2) based on neighbor-joining method also indicated its closest similarity to *Streptomyces parvulus*. The phenotypic and genomic data indicated that the strain HUST012 represented a strain of the genus *Streptomyces* for which the strain was referred to as *Streptomyces* sp. strain HUST012.

### Antimicrobial and cytotoxic effects of strain HUST012

The culture filtrate of strain HUST012 exhibited antibacterial activity against all tested Gram positive and Gram negative bacterial strains. The maximum activity was found against MRSE ATCC 35984 (inhibition zone of 35 mm diameter), followed by *A. hydrophila* ATCC 7966 (26 mm) and MSSA ATCC 29213 (25.80 mm). The detailed antimicrobial profiles are shown in Table 1.

**Table 1:** **Antimicrobial activities of the strain *Streptomyces* sp. HUST012 against bacterial and fungal strains**.

**Test strains**	**Inhibition zone (mm diameter)**
**GRAM POSITIVE BACTERIA**	
Methicillin-resistant *S. epidermidis* ATCC 35984	35.00 ± 0.80
Methicillin-resistant *S. aureus* ATCC 25923	18.90 ± 1.67
Methicillin-susceptible *S. aureus* ATCC 29213	25.80 ± 1.47
*Enterococcus faecalis* ATCC 29212	20.00 ± 0.20
**GRAM NEGATIVE BACTERIA**	
*Escherichia coli* ATCC 25922	18.90 ± 1.00
*Escherichia coli* ATCC 11105	12.40 ± 0.73
*Klebsiella pneumoniae* ATCC 13883	19.80 ± 2.20
*Aeromonas hydrophila* ATCC 7966	26.00 ± 0.47
**FUNGAL STRAINS**	
*Fusarium graminearum*	9.70 ± 0.73
*Aspergillus westerdijkiae*	1.80 ± 0.47
*Aspergillus carbonarius*	7.70 ± 0.80

The antifungal activity of *Streptomyces* sp. strain HUST012 was examined against three mycotoxin producing fungal strains. The fungal growth inhibition was observed in the order: *F. graminearum* (9.7 mm), *A. carbonarius* (7.7 mm), and *A. westerdijkiae* (1.8 mm).

### Fermentation, antimicrobial and cytotoxic effects of bioactive metabolites of strain HUST012

Among the crude metabolites extracts of strain HUST012, the fraction SP-E showed the highest antibacterial and cytotoxic activities. This fraction was further purified by column chromatography with different gradient solvent systems as depicted in Figure 1. Two bioactive metabolites, designated SPE-B11.8 and SPE-B5.4, were purified.

The MIC values of the metabolite SPE-B11.8 against the test bacterial organisms ranges between 15.63 and 62.5 μg/ml while those for SPE-B5.4 ranges between 0.04 and 2.24 μg/ml (Table 2).

**Table 2:** **Antibacterial and cytotoxic effects of the compounds HPE-B11.8 and SPE-B5.4 in comparison with Dragon's blood extracted from medicinal plant *D. cochinchinensis* Lour**.

**Test organisms/cancer cell line**	**SPE-B11.8**	**SPE-B5.4**	**Dragon's blood extract**
	**MIC/IC_50_ (μg/ml)**		
MRSE ATCC 35984	15.63 ± 1.18	0.04 ± 0.00	4.88 ± 0.05
MRSA ATCC 25923	62.5 ± 2.26	0.04 ± 0.00	4.88 ± 0.05
*E. coli* ATCC 25922	Inactive	2.24 ± 0.01	9.77 ± 0.23
*K. pneumoniae* ATCC 13883	62.5 ± 2.26	0.04 ± 0.00	4.88 ± 0.05
*F. graminearum*	Inactive	9.77 ± 0.23	19.53 ± 0.80
Hep G2	41.63 ± 0.61	0.23 ± 0.05	77.91 ± 0.22
MCF-7	29.54 ± 2.89	0.18 ± 0.05	70.00 ± 7.08

Human hepatocellular carcinoma Hep G2 and human breast adenocarcinoma cell MCF-7 lines were used as model systems to examine the cytotoxic effect of *Streptomyces* sp. HUST012. The culture filtrate, crude metabolite extracts (SP-E, SP-W1, SP-W2) and the pure metabolites (SPE-B11.8 and SPE-B5.4) were examined for their cytotoxic effect on the two human cancer cell lines Hep G2 and MCF-7. The cytotoxic assay results showed that the culture filtrate of the strain HUST012 had significant inhibition toward Hep G2 and MCF-7 cells with I*C*_50_-values of 4 and 3 μg/ml, respectively. Among the crude metabolites extracts, SP-E showed the strongest cytotoxic effect with I*C*_50_-values of 0.31 and 0.18 μg/ml. The pure metabolites SPE-B11.8 showed cytotoxic effect at 41.63 and 29.54 μg/ml I*C*_50_-values against Hep G2 and MCF-7, respectively, while the metabolite SPE-B5.4 exhibited the same at 0.23 and 0.18 μg/ml I*C*_50_-values (Table 3).

**Table 3:** **Cytotoxicity of test sample (IC_50_ in μg/ml)**.

**Samples**	**Hep G2**	**MCF-7**
Culture filtrate of HUST012	4.00 ± 0.10	3.00 ± 0.10
SP-E	0.31 ± 0.03	0.18 ± 0.02
SP-W1	>100	>100
SP-W2	>100	>100
SPE-B11.8	41.63 ± 0.61	29.54 ± 2.89
SPE-B 5.4	0.23 ± 0.05	0.18 ± 0.05
Ellipticine	0.51 ± 0.08	0.47 ± 0.05

### Structure elucidation of bioactive compounds

The structure of the compounds SPE-B11.8 and SPE-B5.4 were analyzed through the techniques of MS and NMR spectroscopy.

**Figure 3:**
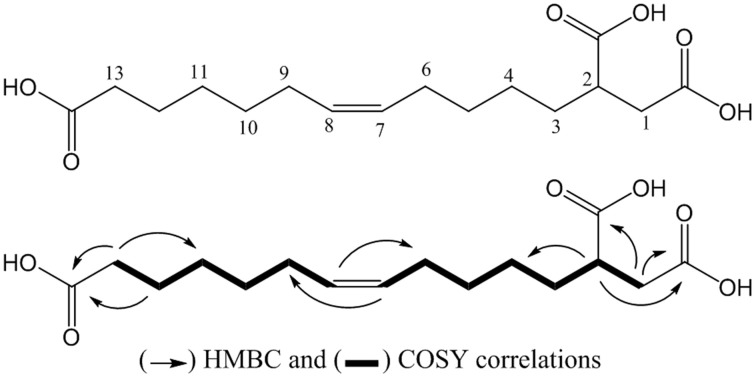
**Structure of the compound SPE-B11.8 (elucidated as (*Z*)-tridec-7-ene-1,2,13-tricarboxylic acid)**.

The compound **SPE-B11.8** was obtained as a colorless solid. Its HRESIMS spectrum showed a peak at *m/*z 315.1814 [M+H]^+^, corresponding to the molecular formula C_16_H_27_O_6_. The 1D and 2D-NMR spectra of **SPE-B11.8** showed signals characteristic for a monounsaturated fatty acid with the double bond at δ_H_ 5.33/δ_C_129.1–129.9, three carboxylic groups at δ_C_ 175.1, 178.1, and 181.1, and a cluster of methylenic protons at δ_C_ in the range of δ_C_ 24.6–35.1. The COSY and HMBC spectra led to the identification of the fragments of SPE-B11.8 structure (see Figure S2 for the complete NMR spectra). The position of the double bond was also confirmed by the MS data with the fragment at *m/z* 128.08 and 187.08 corresponding to the breakdown at C-7 and C-8 liason. The configuration of the double bond was determined based the on the chemical shifts of vicinal carbon atoms. Both C-6 and C-9 appeared at δ_C_ 26.5 and 26.3 ppm indicating the *Z* configuration. Thus, compound SPE-B11.8 was newly elucidated to be (*Z*)-tridec-7-ene-1,2,13-tricarboxylic acid (Figure 3).

**Figure 4:**
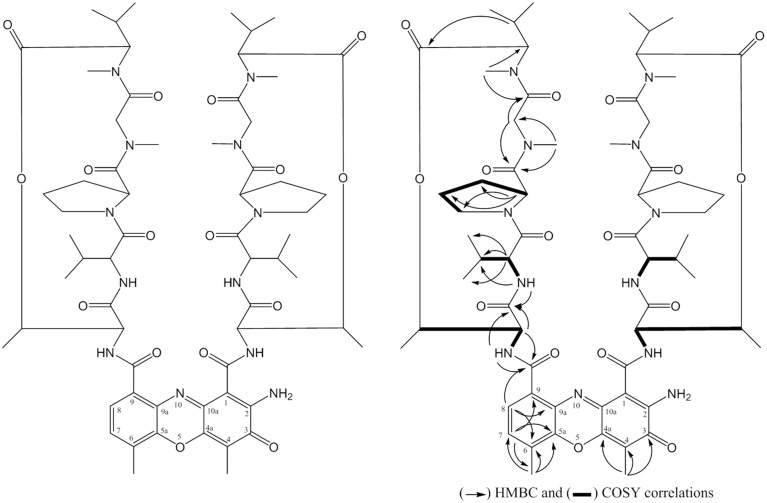
**Structure of the compound SPE-B3.4 (established as Actinomycin-D)**.

The compound **SPE-B5.4** was obtained as a red powder, soluble in methanol, ethyl acetate, ethanol, and DMSO, stable in aqueous solutions at 5–10°C. The HRESIMS spectrum revealed a peak at *m/z* 1255.6435 [M+H]^+^, corresponding to the formula C_62_H_87_N_12_O_16_ (Figure S3). The ^1^H-NMR, ^13^C-NMR spectrum analysis data of the SPE-B5.4 compound is presented in Table S1. The spectral data was compared with the findings of Booth et al. (1976) and the compound SPEB-5.4 was identified as Actinomycin-D with molecular formula C_62_H_86_N_12_O_16_ (Figure 4).

### Antibacterial and cytotoxic effects of the dragon's blood extracted from medicinal plant *D. cochinchinensis*

The Dragon's blood extract was analyzed for its antibacterial and cytotoxic effects against MRSA, MRSE, *K. pneumoniae* and *E. coli*, and toward MCF-7 and Hep G2 cell lines. Table 2 showed the MIC for the dragon's blood extracts in comparison with those of the crude metabolites extracts and the compounds SPE-B11.8 and SPE-B5.4.

## Discussion

The antimicrobial resistance has been one of the most serious health threats. Infections from resistant bacteria are now too common, and some pathogens have even become resistant to multiple classes of antibiotics. The decline of effective antibiotics will undermine our ability to fight infectious diseases and manage the infectious complications common in vulnerable patients, especially those undergoing chemotherapy for cancer, dialysis for renal failure, and organ transplantation. When first- and second-line antibiotic treatment options are limited by resistance and/or unavailability, healthcare providers are forced to use toxic antibiotics which are frequently more expensive but less effective. Even when alternative treatments are available, research has shown that patients with resistant infections are often much more likely to result in death, and that survivors require longer hospital stays, delayed recuperation, and long-term disability. Hence, there is an urgent need for search of novel drugs against such pathogens. It has been envisaged that endophytic environment is an extreme source to provide exciting new bioactive compounds.

In the present study, an attempt was tried to identify the bioactive potential of the endophytic actinobacterium *Streptomyces* sp. HUST012. The strain was found to exhibit antimicrobial activities against a set of pathogenic bacteria and fungi (Table 1). The presence of antifungal activities is also an indication of probable biocontrol mechanisms against mycotoxin producing fungal strains. Similar findings have been reported in similar studies of *Streptomyces* strains (Rahman et al., 2010; Usha et al., 2010; Naine et al., 2015).

The cytotoxic ability of this strain was significant as compared to that reported in previous studies on *S. parvulus* strain VITJS11 (Naine et al., 2015). The compounds SPE-B11.8 and SPE-B5.4 had I*C*_50_-values of 41.63 and 0.23 μg/ml on Hep G2 cells as compared to 500 μg/ml by *S. parvulus* strain VITJS11. Other reports showed that migrastatin, a secondary metabolite from *Streptomyces* inhibited the Hep G2 cells at the concentration of 6 and 10 μM after 24 and 48 h of treatment (Rambabu et al., 2014). The high bioactive effect of *Streptomyces* sp. HUST012 can be explained by the fact that endophytic actinomycetes live in close association with their host plants and that it could become a real possibility for exchange of genes involved in natural products biosynthesis between endophytic actinomycetes and host plants via horizontal gene transfer, resulting in synthesis of plant-derived compounds by a microbial endophyte (Chandra et al., 2013).

An important finding of this current study was the isolation of the new compound HPE-B11.8 which was elucidated as (*Z*)-tridec-7-ene-1,2,13-tricarboxylic acid, thereby underlying the importance of the source. The compound HPE-B11.8 possessed moderate antibacterial and anticancer activities against the test pathogenic microorganisms/cell lines. Another important finding was the isolation of Actinomycin D (compound SPE-B5.4). Actinomycin D was an antineoplastic antibiotic that inhibits cell proliferation. It finds wide range of applications, viz. as selective reagent in cell culture, studies in suppressing HIV-replication and programmed cell death of PC12 cells, and as an antibiotic in treatment of various malignant neoplasm including Wilm's tumor and the sarcomas. Actinomycin-D decreases Mcl-1 expression and acts synergistically with ABT-737 against small cell lung cancer cell lines (Aishan et al., 2010). According to the Internet bibliographic database-MEDLINE, actinomycins, especially Actinomycin-D, have been the subject of about 3300 research publications (Koba and Konopa, 2005). The isolation of Actinomycin-D is not a new discovery but our present study proved that the medicinal plant *D. cochinchinensis* Lour. was a rich source of endophytic actinomycetes producing the potent antibiotic agents.

Dragon's blood has been well documented for its antimicrobial, antioxidant, anti-antitumor and cytotoxic properties. However, the host plant *D. cochinchinensis* Lour. has no secretory tissue to release this useful metabolite, and therefore Dragon's blood remains in xylem parenchyma cells of the stem. The growth of the plant is extremely slow and has low yield of dragon's blood. To harvest a few pieces of resinous wood, a tree with hundreds of years old is often destroyed. This work aimed to evaluate the antimicrobial and cytotoxic effects of the natural Dragon's blood extracted from medicinal plant *D. cochinchinensis* Lour. in comparison with that secreted by the endophytic *Streptomyces* sp. HUST012 associated with the host plant. Our results were significant in comparison to the findings of other research groups (Al-Fatimi et al., 2005; Wang et al., 2010, 2011). This could give us a suggestion for the promotion of the application of secondary bioactive metabolites from endophytic actinomycetes associated with the medicinal plant *D. cochinchinensis* Lour. instead of destroying valuable endangered trees.

### Conflict of interest statement

The authors declare that the research was conducted in the absence of any commercial or financial relationships that could be construed as a potential conflict of interest.

## References

[B1] Aishan H. Baba M. Iwasaki N. Kuang H. Okuyama T. (2010). The constituents of *Urtica cannabina* used in Uighur medicine. Pharm. Biol. 48, 577–583. 10.3109/13880200903214215 20645802

[B2] Al-Fatimi M. Friedrich U. Jenett-Siems K. (2005). Cytotoxicity of plants used in traditional medicine in Yemen. Fitoterapia 76, 355–358. 10.1016/j.fitote.2005.02.009 15890471

[B3] Anderson A. S. Wellington E. M. (2001). The taxonomy of *Streptomyces* and related genera. Int. J. Syst. Evol. Microbiol. 51, 797–814. 10.1099/00207713-51-3-797 11411701

[B4] Andrews J. M. (2001). Determination of minimum inhibitory concentrations. J. Antimicrob. Chemother. 48, 5–16. 10.1093/jac/48.suppl_1.5 11420333

[B5] Athalye M. Goodfellow M. Lacey J. White R. P. (1985). Numerical classification of *Actinomadura* and *Nocardiopsis*. Int. J. Syst. Bact. 35, 86–98. 10.1099/00207713-35-1-86

[B6] Booth H. Mauger A. B. Rzeszotarski W. J. (1976). A ^13^C NMR study of actinomycin D and related model peptides. Org. Magn. Reson. 8, 219–223. 10.1002/mrc.1270080413

[B7] Chandra S. Lata H. Varma A. (2013). Biotechnology for Medicinal Plants-Micropropagation and Improvement. Berlin; Heidelberg: Springer-Verleg.

[B8] Crnovcic I. Vater J. Keller U. (2013), Occurrence and biosynthesis of C-demethylactinomycins in actinomycin-producing *Streptomyces chrysomallus* and *Streptomyces parvulus*. J. Antibiot. 66, 211–218. 10.1038/ja.2012.120 23423168

[B9] Fan L. L. Tu P. F. He J. X. Chen H. B. Cai S. Q. (2008). Microscopical study of original plant of Chinese drug “Dragon's Blood” *Dracaena cochinchinensis* and distribution and constituents detection of its resin. Zhongguo Zhong Yao Za Zhi 33, 1112–1117. 18720856

[B10] Felsenstein J. (1985). Confidence limits on phylogenies: an approach using the bootstrap. Evolution 39, 783–791. 10.2307/2408678 28561359

[B11] Goodfellow M. (1971). Numerical taxonomy of some nocardioform bacteria. J. Gen. Microbiol. 69, 33–80. 10.1099/00221287-69-1-33 4948190

[B12] Gopal R. Na H. Seo C. H. Park Y. (2012). Antifungal activity of (KW)n or (RW)n peptide against *Fusarium solani* and *Fusarium oxysporum*. Int. J. Mol. Sci. 13, 15042–15053. 10.3390/ijms131115042 23203110 PMC3509626

[B13] Gupta D. Bleakley B. Gupta R. K. (2008). Dragon's blood: botany, chemistry and therapeutic uses. J. Ethnopharmacol. 115, 361–380. 10.1016/j.jep.2007.10.018 18060708

[B14] Hamza A. A. Ali H. A. Clark B. R. Murphy C. D. Elobaid E. A. (2013). Isolation and characterisation of actinomycin D producing *Streptomyces* spp. from Sudanese soil. Afr. J. Biotechnol. 12, 2624–2632. 10.5897/AJB12.1066

[B15] Holder I. A. Boyce S. T. (1994). Agar well diffusion assay testing of bacterial susceptibility to various antimicrobials in concentrations non-toxic for human cells in culture. Burns 20, 426–429. 10.1016/0305-4179(94)90035-3 7999271

[B15a] Huffman J. Gerber R. Du L. (2010). Recent advancements in the biosynthetic mechanisms for polyketide-derived mycotoxins. Biopolymers 93, 764–776. 10.1002/bip.21483 20578001 PMC2894268

[B16] Inbar L. Lapidot A. (1988). Metabolic regulation in *Streptomyces parvulus* during actinomycin D synthesis, studied with 13C- and 15N-labeled precursors by 13C and 15N nuclear magnetic resonance spectroscopy and by gas chromatography-mass spectrometry. J. Bacteriol. 170, 4055–4064. 3410824 10.1128/jb.170.9.4055-4064.1988 PMC211409

[B17] Kawahara T. Izumikawa M. Otoguro M. Yamamura H. Hayakawa M. Takagi M.. (2012). JBIR-94 and JBIR-125, Antioxidative phenolic compounds from *Streptomyces* sp. R56-07. J. Nat. Prod. 75, 107–110. 10.1021/np200734p 22233425

[B18] Khamna S. Yokota A. Lumyong S. (2009). Actinomycetes isolated from medicinal plant rhizospheric soils: diversity and screening of antifungal compounds, indole-3-acetic acid and siderophore production. World J. Microbiol. Biotechnol. 25, 649–655. 10.1007/s11274-008-9933-x

[B19] Kim O. S. Cho Y. J. Lee K. Yoon S. H. Kim M. Na H.. (2012). Introducing EzTaxon-e: a prokaryotic 16S rRNA gene sequence database with phylotypes that represent uncultured species. Int. J. Syst. Evol. Microbiol. 62, 716–721. 10.1099/ijs.0.038075-0 22140171

[B20] Kimura M. (1983). The Neutral Theory of Molecular Evolution. Cambridge: Cambridge University Press.

[B21] Koba M. Konopa J. (2005). Actinomycin D and its mechanisms of action. Postepy Hig. Med. Dosw. 59, 290–298. 15995596

[B22] Koup J. R. Brodsky B. Lau A. Beam T. R. Jr. (1978). High-performance liquid chromatographic assay of chloramphenicol in serum. Antimicrob. Agents Chemother. 14, 439–443. 708020 10.1128/aac.14.3.439 PMC352477

[B23] Li J. Zhao G. Z. Qin S. Zhu W. Y. Xu L. H. Li W. J. (2009). *Streptomyces sedi* sp. nov., isolated from surface-sterilized roots of *Sedum* sp. Int. J. Syst. Evol. Microbiol. 59, 1492–1496. 10.1099/ijs.0.007534-0 19502341

[B24] Liu Y. Tortora G. Ryan M. E. Lee H. M. Golub L. M. (2002). Potato dextrose agar antifungal susceptibility testing for yeasts and molds: evaluation of phosphate effect on antifungal activity of CMT-3. Antimicrob. Agents Chemother. 46, 1455–1461. 10.1128/AAC.46.5.1455-1461.2002 11959582 PMC127172

[B25] Mechri B. Manga A. G. B. Tekaya M. Attia F. Cheheb H. Meriem F. B. (2014). Changes in microbial communities and carbohydrate profiles induced by the *mycorrhizal* fungus (*Glomus intraradices*) in rhizosphere of *olive* trees (*Olea europaea* L.). Appl. Soil Ecol. 75, 124–133. 10.1016/j.apsoil.2013.11.001

[B26] Naine S. J. Devi C. S. Mohanasrinivasan V. Vaishnavi B. (2015). Antimicrobial, antioxidant and cytotoxic activity of marine *Streptomyces parvulus* VITJS11 crude extract. Braz. Arch. Biol. Technol. 58, 198–207. 10.1509/S1516-8913201400173

[B27] Olano C. Moss S. J. Braña A. F. Sheridan R. M. Math V. Weston A. J.. (2004). Biosynthesis of the angiogenesis inhibitor borrelidin by *Streptomyces parvulus* Tü4055: insights into nitrile formation. Mol. Microbiol. 52, 1745–1756. 10.1111/j.1365-2958.2004.04090.x 15186422

[B28] Pridham T. G. Gottlieb D. (1948). The utilization of carbon compounds by some *Actinomycetales* as an aid for species determination. J. Bacteriol. 56, 107–114. 16561537 10.1128/jb.56.1.107-114.1948 PMC518551

[B29] Qin S. Li J. Chen H. H. Zhao G. Z. Zhu W. Y. Jiang C. L.. (2009). Isolation, diversity, and antimicrobial activity of rare actinobacteria from medicinal plants of tropical rain forests in Xishuangbanna, China. Appl. Environ. Microbiol. 75, 6176–6186. 10.1128/AEM.01034-09 19648362 PMC2753051

[B30] Qin S. Xing K. Jiang J. H. Xu L. H. Li W. J. (2011). Biodiversity, bioactive natural products and biotechnological potential of plant-associated endophytic actinobacteria. Appl. Microbiol. Biotechnol. 89, 457–473. 10.1007/s00253-010-2923-6 20941490

[B31] Rahman M. A. Islam M. Z. Khondkar P. Islam M. A. U. (2010). Characterization and antimicrobial activities of a polypeptide antibiotic isolated from a new strain of *Streptomyces parvulus*. Bangladesh Pharm. J. 13, 14–17.

[B32] Rambabu V. Suba S. Manikandan P. Vijayakumar S. (2014). Cytotoxic and apoptotic nature of migrastatin, a secondary metabolite from *Streptomyces* evaluated on HEP G2 cell line. Int. J. Pharm. Pharm. Sci. 6, 333–338.

[B33] Rehm S. Han S. Hassani I. Sokocevic A. Jonker H. R. Engels J. W.. (2009). The high resolution NMR structure of parvulustat (Z-2685) from *Streptomyces parvulus* FH-1641: comparison with tendamistat from *Streptomyces tendae* 4158. Chembiochem 10, 119–127. 10.1002/cbic.200800547 19067455

[B34] Saitou N. Nei M. (1987). The neighbour-joining method: a new method for reconstructing phylogenetic trees. Mol. Biol. Evol. 4, 406–425. 3447015 10.1093/oxfordjournals.molbev.a040454

[B35] Shirling E. B. Gottlieb D. (1966). Methods for characterization of *Streptomyces* species. Int. J. Syst. Bacteriol. 16, 313–340. 10.1099/00207713-16-3-313

[B36] Subramani R. Aalbersberg W. (2012). Marine actinomycetes: an ongoing source of novel bioactive metabolites. Microbiol. Res. 167, 571–580. 10.1016/j.micres.2012.06.005 22796410

[B37] Tamura K. Stecher G. Peterson D. Filipski A. Kumar S. (2013), MEGA6: Molecular Evolutionary Genetics Analysis version 6.0. Mol. Biol. Evol. 30, 2725–2729. 10.1093/molbev/mst197 24132122 PMC3840312

[B38] Thao D. T. Phuong D. T. Hanh T. T. H. Thao N. P. Cuong N. X. Nam N. H.. (2014). Two new neoclerodane diterpenoids from Scutellaria barbata D. Don growing in Vietnam. J. Asian Nat. Prod. Res. 16, 364–369. 10.1080/10286020.2014.882912 24498964

[B39] Thompson J. D. Gibson T. J. Plewniak F. Jeanmougin F. Higgins D. G. (1997). The CLUSTAL_X windows interface: flexible strategies for multiple sequence alignment aided by quality analysis tools. Nucleic Acids Res. 25, 4876–4882. 10.1093/nar/25.24.4876 9396791 PMC147148

[B40] Tindall B. J. Sikorski J. Smibert R. A. Krieg N. R. (2007). Phenotypic characterization and the principles of comparative systematics, in Methods for General and Molecular Microbiology, 3rd Edn., eds Reddy C. A. Beveridge T. J. Breznak J. A. Marzluf G. A. Schmidt T. M. Snyder L. R. (Washington, DC: ASM Press), 330–393. 10.1128/9781555817497.ch15

[B41] Usha R. Ananthaselvi P. Venil C. K. Palaniswamy M. (2010). Antimicrobial and antiangiogenesis activity of *Streptomyces parvulus* KUAP106 from Mangrove Soil. Eur. J. Biol. Sci. 2, 77–83.

[B42] Wang X. H. Zhang C. Yang L. L. Gomes-Laranjo J. (2011). Production of dragon's blood in *Dracaena cochinchinensis* plants by inoculation of *Fusarium proliferatum*. Plant Sci. 180, 292–299. 10.1016/j.plantsci.2010.09.007 21421373

[B43] Wang X. H. Zhang C. H. Wang Y. Gomes-Laranjo J. (2010). Screen of micro-organisms for inducing the production of dragon's blood by leaf of *Dracaena cochinchinensis*. Lett. Appl. Microbiol. 51, 504–510. 10.1111/j.1472-765X.2010.02921.x 20860774

[B44] Xu P. Li W. J. Tang S. K. Zhang Y. Q. Chen G. Z. Chen H. H.. (2005). *Naxibacter alkalitolerans* gen. nov., sp. nov., a novel member of the family 'Oxalobacteraceae' isolated from China. Int. J. Syst. Evol. Microbiol. 55, 1149–1153. 10.1099/ijs.0.63407-0 15879247

[B45] Zhang Y. Q. Liu H. Y. Chen J. Yuan L. J. Sun W. Zhang L. X.. (2010). Diversity of culturable actinobacteria from Qinghai-Tibet plateau, China. Antonie Van Leeuwenhoek 98, 213–223. 10.1007/s10482-010-9434-4 20361256

