# Supplementary material for: Characterization and evaluation of antimicrobial and cytotoxic effects of Streptomyces sp. HUST012 isolated from medicinal plant Dracaena cochinchinensis Lour
Source: Front Microbiol. 2015 Jun 8;6:574. doi: 10.3389/fmicb.2015.00574 (PMC4458686; doi:10.3389/fmicb.2015.00574)
Supplement: Supplementary file 1 [file Table1.PDF]

Supplementary Material

Characterization and evaluation of antimicrobial and cytotoxic effects of *Streptomyces* sp. HUST012 isolated from medicinal plant *Dracaena cochinchinensis* Lour.

Thi-Nhan Khieu[1,2], Min-Jiao Liu[1,3], Salam Nimaichand[4], Ngoc-Tung Quach[5], Son Chu-Ky[2], Quyet-Tien Phi[5], Thu-Trang Vu[2], Tien-Dat Nguyen[6], Zhi Xiong[3], Deene Manik Prabhu[1], Wen-Jun Li[1,4]*

[1]*Key Laboratory of Microbial Diversity in Southwest China, Ministry of Education, Yunnan Institute of Microbiology, Yunnan University, Kunming, P. R. China,*
[2]*Department of Food Technology, School of Biotechnology and Food Technology, Hanoi University of Science and Technology, Hanoi, Vietnam,*
[3]*Key Laboratory for Forest Resources Conservation and Use in the Southwest Mountains of China, Ministry of Education, Southwest Forestry University, Kunming 650224, PR China,*
[4]*State Key Laboratory of Biocontrol, Key Laboratory of Biodiversity Dynamics and Conservation of Guangdong Higher Education Institutes, College of Ecology and Evolution, Sun Yat-Sen University, Guangzhou, PR China,*
[5]*Laboratory of Fermentation Technology, Institute of Biotechnology, Vietnam Academy of Science and Technology, Hanoi, Vietnam,*
[6]*Department of Bioactive Products, Institute of Marine Biochemistry, Vietnam Academy of Science and Technology, Hanoi, Vietnam*

Supplementary Table S1. Spectral analysis data for D-actinomycin and compound SPE-B5.4

	Actinomycin D (Booth et al., 1976)		Compound SPE-B5.4			
	α-ring	β-ring	α-ring		β-ring	
	δ_C	δ_C	δ_C	δ_H	δ_C	δ_H
(1)	(2)	(3)	(4)	(5)	(6)	(7)
L-Meval						
C=O	#	#	167.59		167.68	
α-CH	71.3/71.4		71.28 (71.45)	2.68, m	71.28 (71.45)	2.68, m
β-CH	26.9	26.9	26.92	2.67, m	26.92	2.67, m
$N-CH_3$	39.2	39.2	39.20	2.90, m	39.20	2.90, m

			(39.32)	(2.93, m)	(39.32)	(2.93, m)
$2xCH_3$	&	&	@	$	@	$
Sar						
C=O	#	#	173.74		173.74	
CH_2	51.4	51.4	51.39	3.62, m<br>4.78, m	51.39	3.62, m<br>4.78, m
$N-CH_3$	34.9	34.9	34.87<br>(34.93)	2.88, m	34.87<br>(34.93)	2.88, m
L-Pro						
C=O	#	#	173.31<br>(173.36)		173.31<br>(173.36)	
α-CH	56.3	56.3	56.25	6.03 d (9.0)	56.43	5.96 d (9.0)
β-CH	31.0/31.3		31.56	2.16, m	31.83	2.18, m
$\gamma-CH_2$	22.9/23.1		23.04	2.27, m<br>2.09, m	22.88	2.27, m<br>2.09, m
$\delta-CH_2$	47.3/47.5		47.35	3.98, m<br>3.72, m	47.61	3.83, m<br>3.73, m
D-Val						
C=O	#	#	166.21		166.21	
α-CH	58.7	58.7	58.74	3.54, m	58.90	3.56, m
β-CH	31.5/31.7		31.83	2.18, m	31.56	2.16, m
$2xCH_3$	&	&	@	$	@	$
L-Thr						
C=O	#	#	168.51		168.98	
α-CH	54.9/55.3		55.27	4.51 dd(2.5, 7.0)	54.90	4.62 dd(2.5, 6.5)
β-CH	75.0	75.0	75.00	5.21 dd(2.5, 6.5)	75.07	5.19 dd(2.0, 6.4)
CH_3	17.8	17.4	17.82	1.25, s	17.40	1.25, s
Phenoxazone						
	Actinomycin D		Compound SPE-B5.4			
	δ_C		δ_C		δ_H	

C=O	#	166.34/166.55	
1	101.6	101.70	
2	146.0	145.90	
3	#	179.12	
4	113.5	113.53	
4a	145.1	145.12	
5a	140.5	145.50	
6	127.5	127.63	
7	130.2	130.29	7.37, d (8.0)
8	125.9	125.85	7.64, d (7.5)
9	132.6	132.63	
9a	129.1	129.12	
10a	147.6	147.67	
4-CH_3	7.7	7.78	2.24, s
6-CH_3	15.0	15.05	2.55, s

#: 13 "C = O" groups of actinomycin D (Booth et al., 1976) δ_C = 166,1; 166,3; 2 x 166,5; 2 x 167,6; 168,5; 168,9; 3 x 173,3; 173,7 và 179,1.

&: 8 "CH_3" groups of actinomycin D (Booth et al., 1976) δ_C = 6 x19,1, 2 x 21,6

@: 8 "CH_3" groups of SPEB-5.4 (**1**) measured δ_C = 19,02; 19,07; 19,11; 19,13; 19,26; 19,31; 21,59; 21,69.

$: 8 proton signals of 8 methyl groups at δH = 2 x 1.12; 2 x 0.75; 2 x 0.90; 2 x 0.96.
