# Supplementary material for: Characterization and evaluation of antimicrobial and cytotoxic effects of Streptomyces sp. HUST012 isolated from medicinal plant Dracaena cochinchinensis Lour
Source: Front Microbiol. 2015 Jun 8;6:574. doi: 10.3389/fmicb.2015.00574 (PMC4458686; doi:10.3389/fmicb.2015.00574)

Supplementary Material

Characterization and evaluation of antimicrobial and cytotoxic effects of *Streptomyces* sp. HUST012 isolated from medicinal plant *Dracaena cochinchinensis* Lour.

Thi-Nhan Khieu[1,2], Min-Jiao Liu[1,3], Salam Nimaichand[4], Ngoc-Tung Quach[5], Son Chu-Ky[2], Quyet-Tien Phi[5], Thu-Trang Vu[2], Tien-Dat Nguyen[6], Zhi Xiong[3], Deene Manik Prabhu[1], Wen-Jun Li[1,4]*

[1]*Key Laboratory of Microbial Diversity in Southwest China, Ministry of Education, Yunnan Institute of Microbiology, Yunnan University, Kunming, P. R. China,*
[2]*Department of Food Technology, School of Biotechnology and Food Technology, Hanoi University of Science and Technology, Hanoi, Vietnam,*
[3]*Key Laboratory for Forest Resources Conservation and Use in the Southwest Mountains of China, Ministry of Education, Southwest Forestry University, Kunming 650224, PR China,*
[4]*State Key Laboratory of Biocontrol, Key Laboratory of Biodiversity Dynamics and Conservation of Guangdong Higher Education Institutes, College of Ecology and Evolution, Sun Yat-Sen University, Guangzhou, PR China,*
[5]*Laboratory of Fermentation Technology, Institute of Biotechnology, Vietnam Academy of Science and Technology, Hanoi, Vietnam,*
[6]*Department of Bioactive Products, Institute of Marine Biochemistry, Vietnam Academy of Science and Technology, Hanoi, Vietnam*

*To whom correspondence should be addressed:
Prof. Wen-Jun Li, Key Laboratory of Microbial Diversity in Southwest China, Ministry of Education, Yunnan Institute of Microbiology, Yunnan University, Kunming, 650091, P. R. China; Email: wjli@ynu.edu.cn; liact@hotmail.com

Supplementary Figure S1 a) Growth morphology of HUST012 in ISP2 and YIM 38 media; b) Scanning Electron Micrograph of spore chains, Bar 1 μm and 5 μm

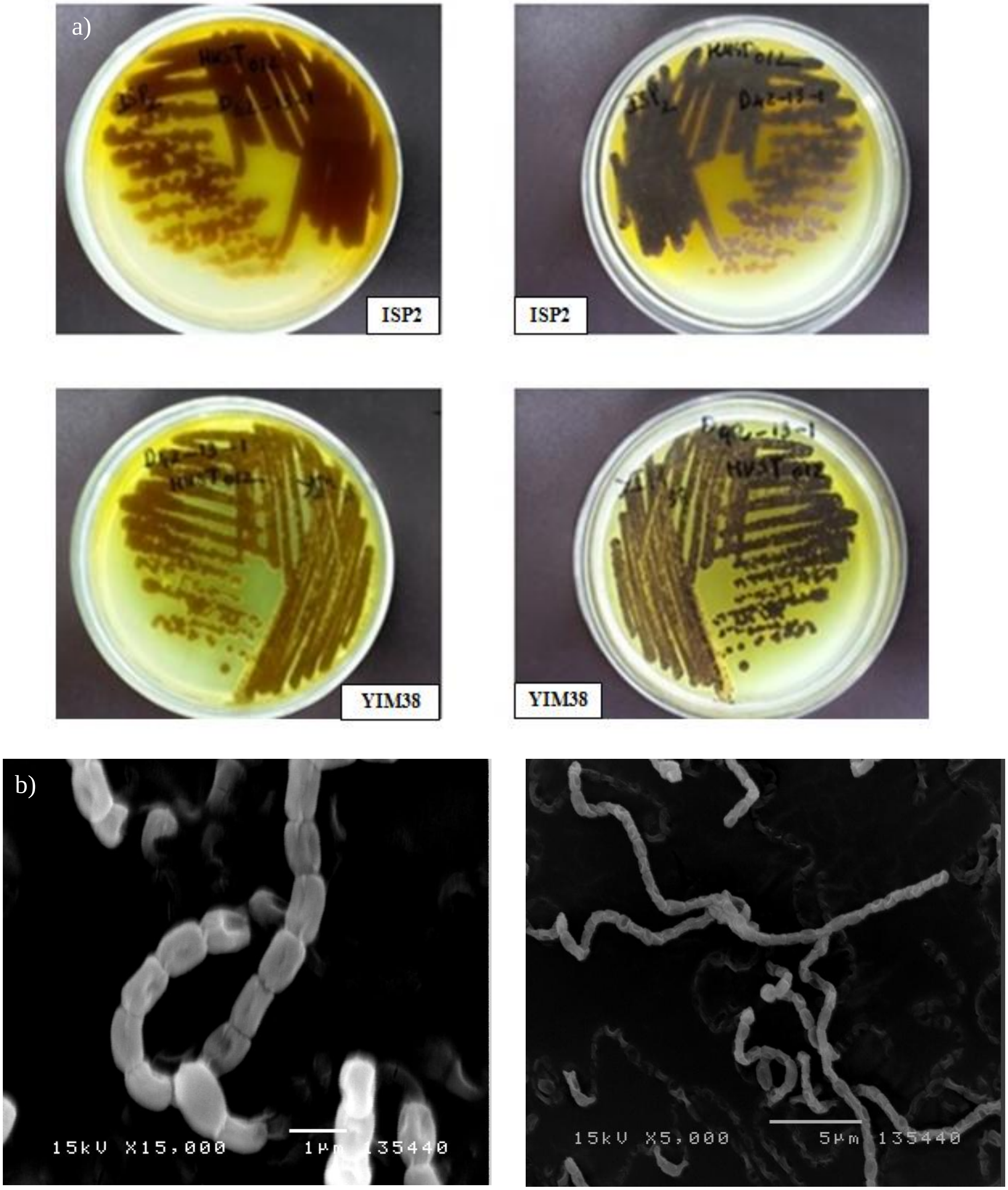

Supplement: Supplementary file 2 [file Image1.PDF]
