# Supplementary material for: Characterization and evaluation of antimicrobial and cytotoxic effects of Streptomyces sp. HUST012 isolated from medicinal plant Dracaena cochinchinensis Lour
Source: Front Microbiol. 2015 Jun 8;6:574. doi: 10.3389/fmicb.2015.00574 (PMC4458686; doi:10.3389/fmicb.2015.00574)
Supplement: Supplementary file 3 [file Image2.PDF]

Supplementary Material

Characterization and evaluation of antimicrobial and cytotoxic effects of *Streptomyces* sp. HUST012 isolated from medicinal plant *Dracaena cochinchinensis* Lour.

Thi-Nhan Khieu[1,2], Min-Jiao Liu[1,3], Salam Nimaichand[4], Ngoc-Tung Quach[5], Son Chu-Ky[2], Quyet-Tien Phi[5], Thu-Trang Vu[2], Tien-Dat Nguyen[6], Zhi Xiong[3], Deene Manik Prabhu[1], Wen-Jun Li[1,4]*

[1]*Key Laboratory of Microbial Diversity in Southwest China, Ministry of Education, Yunnan Institute of Microbiology, Yunnan University, Kunming, P. R. China,* [2]*Department of Food Technology, School of Biotechnology and Food Technology, Hanoi University of Science and Technology, Hanoi, Vietnam,*[3]*Key Laboratory for Forest Resources Conservation and Use in the Southwest Mountains of China, Ministry of Education, Southwest Forestry University, Kunming 650224, PR China,*[4]*State Key Laboratory of Biocontrol, Key Laboratory of Biodiversity Dynamics and Conservation of Guangdong Higher Education Institutes, College of Ecology and Evolution, Sun Yat-Sen University, Guangzhou, PR China,* [5]*Laboratory of Fermentation Technology, Institute of Biotechnology, Vietnam Academy of Science and Technology, Hanoi, Vietnam,* [6]*Department of Bioactive Products, Institute of Marine Biochemistry, Vietnam Academy of Science and Technology, Hanoi, Vietnam*

*To whom correspondence should be addressed:

Prof. Wen-Jun Li, Key Laboratory of Microbial Diversity in Southwest China, Ministry of Education, Yunnan Institute of Microbiology, Yunnan University, Kunming, 650091, P. R. China; Email: wjli@ynu.edu.cn; liact@hotmail.com

Supplementary Figure S2 NMR spectra of compound **SPE-B11.8:** a) ^{1}H NMR spectrum; b) ^{13}C-NMR spectrum; c) DEPT spectrum; d) COSY spectrum; and e) HMBC spectrum

a)

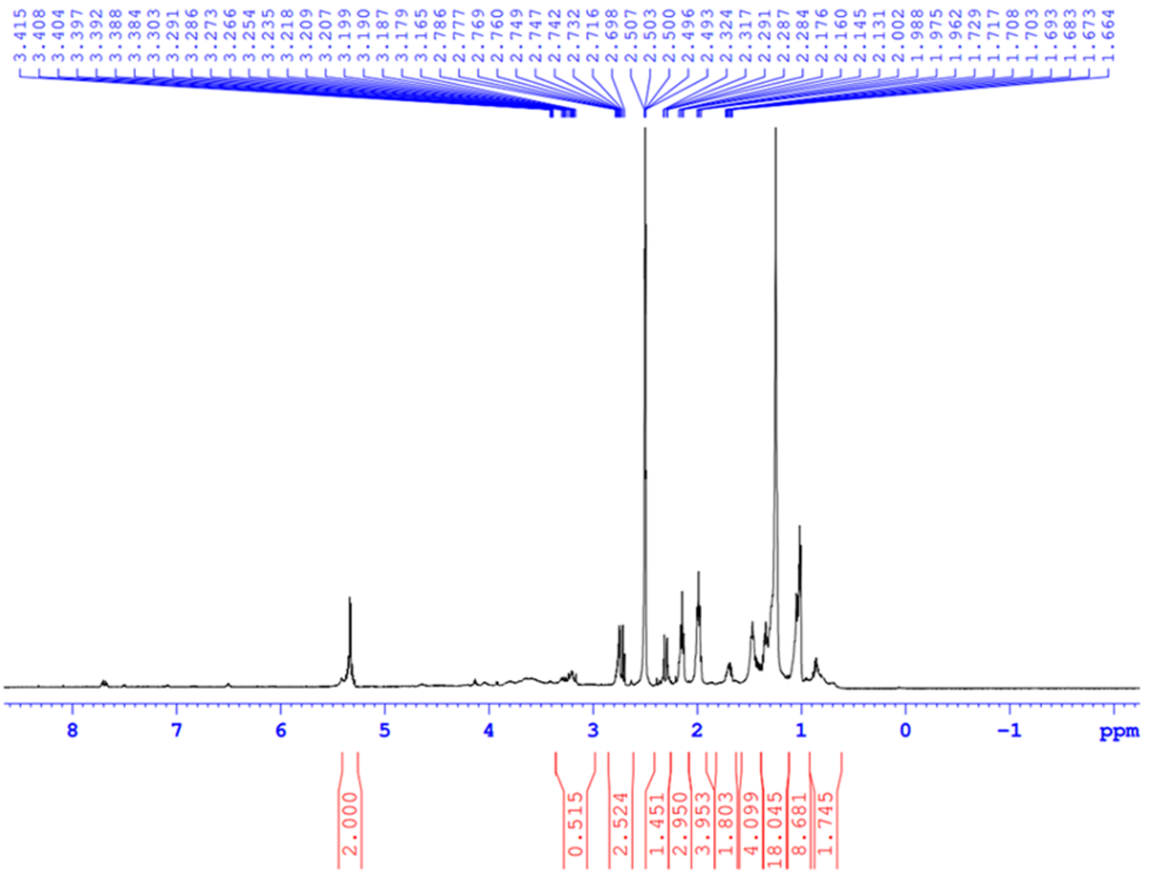

b)

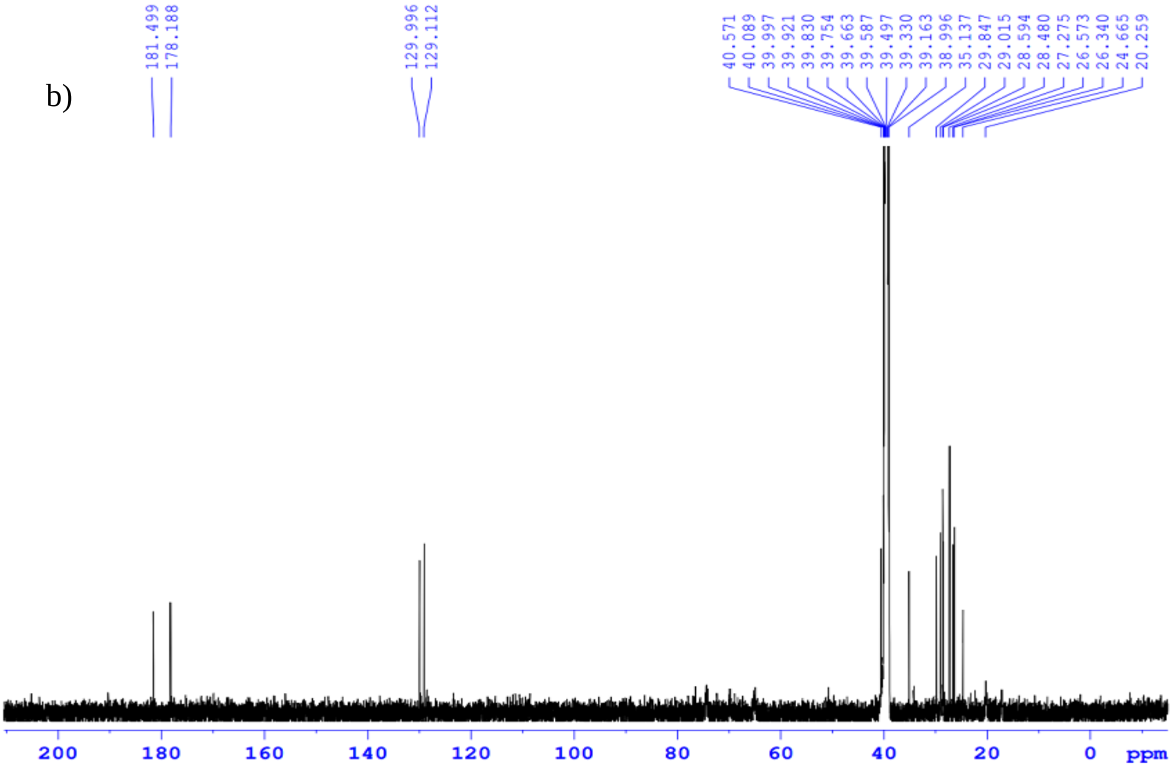

181.499
178.188
129.996
129.112
40.571
40.089
39.997
39.921
39.830
39.754
39.663
39.587
39.497
39.330
39.163
38.996
35.137
29.847
29.015
28.594
28.480
27.275
26.573
26.340
24.665
20.259
200 180 160 140 120 100 80 60 40 20 0 ppm

c)

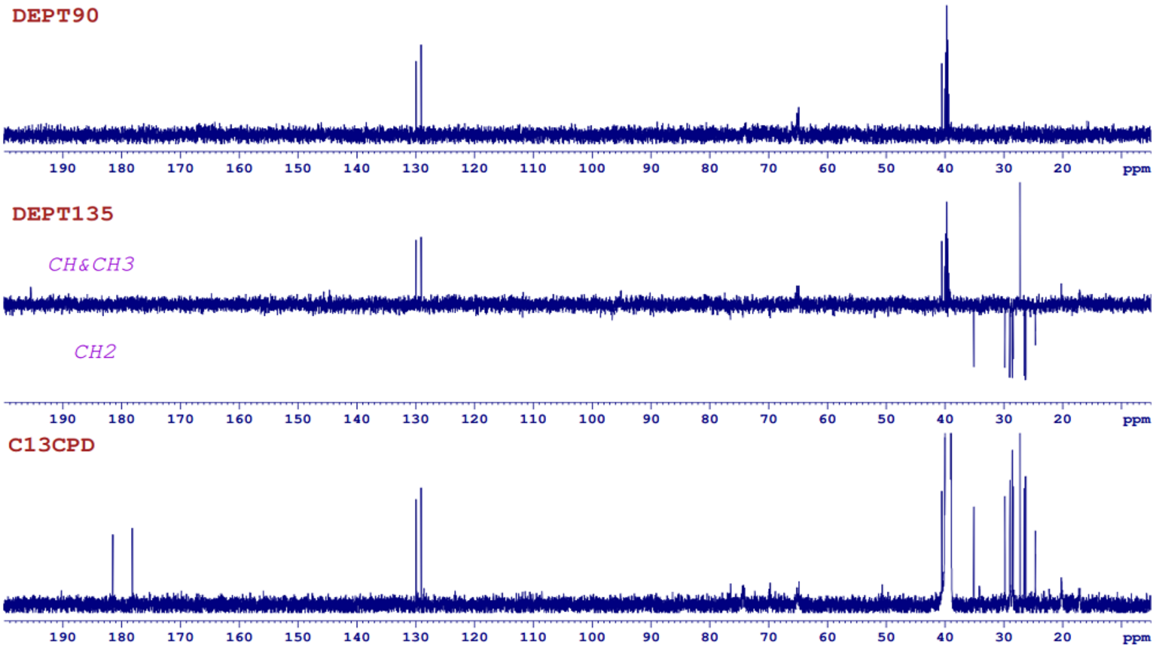

DEPT90
190 180 170 160 150 140 130 120 110 100 90 80 70 60 50 40 30 20 ppm
DEPT135
CH&CH3
CH2
190 180 170 160 150 140 130 120 110 100 90 80 70 60 50 40 30 20 ppm
C13CPD
190 180 170 160 150 140 130 120 110 100 90 80 70 60 50 40 30 20 ppm

d)

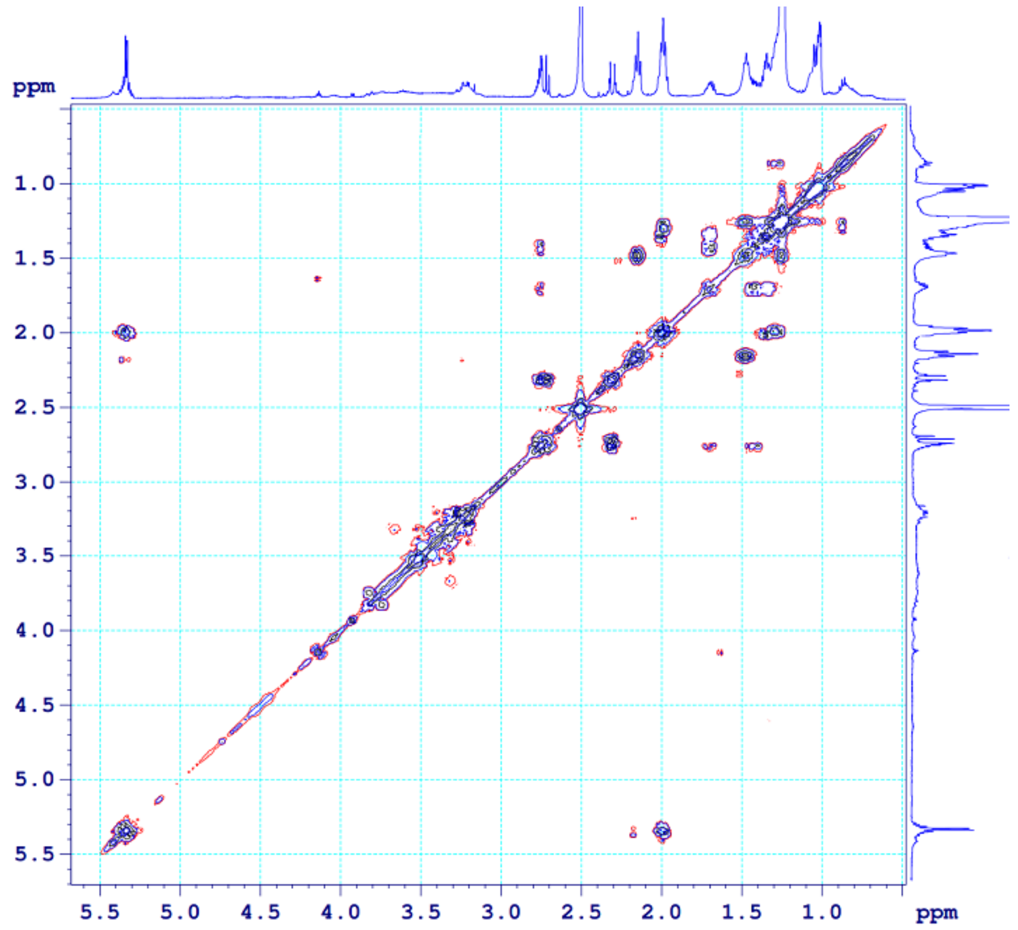
ppm
1.0
1.5
2.0
2.5
3.0
3.5
4.0
4.5
5.0
5.5
5.5 5.0 4.5 4.0 3.5 3.0 2.5 2.0 1.5 1.0
ppm

e)

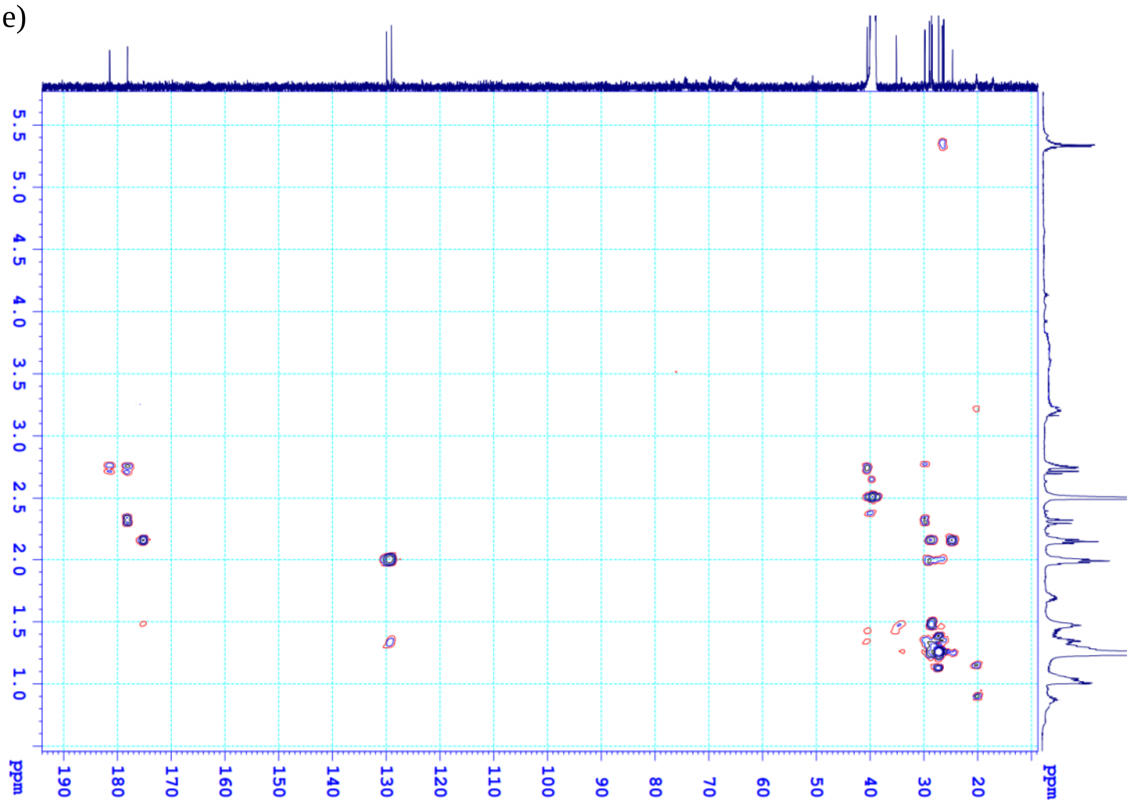
5.5 5.0 4.5 4.0 3.5 3.0 2.5 2.0 1.5 1.0
ppm
190 180 170 160 150 140 130 120 110 100 90 80 70 60 50 40 30 20
ppm
